# Childhood adversity and risk of later labor market marginalization in young employees in Sweden

**DOI:** 10.1093/eurpub/ckad019

**Published:** 2023-02-24

**Authors:** Emma Björkenstam, Magnus Helgesson, Ellenor Mittendorfer-Rutz

**Affiliations:** Division of Insurance Medicine, Department of Clinical Neuroscience, Karolinska Institutet, Stockholm, Sweden; Department of Community Health Sciences, Fielding School of Public Health and California Center for Population Research, University of California Los Angeles, Los Angeles, CA, USA; Department of Medical Sciences, Psychiatry, Uppsala University, Uppsala, Sweden; Division of Insurance Medicine, Department of Clinical Neuroscience, Karolinska Institutet, Stockholm, Sweden; Division of Insurance Medicine, Department of Clinical Neuroscience, Karolinska Institutet, Stockholm, Sweden

## Abstract

**Background:**

The present study examined the independent and combined effects of childhood adversity (CA) and occupational class on the risk of future labor market marginalization (LMM) in young employees in Sweden. Occupational class (non-manual/manual workers) was also explored as a potential mediator.

**Methods:**

This population-based longitudinal cohort study included 556 793 employees, 19–29 years, residing in Sweden in 2009. CAs included parental death, parental mental and somatic disorders, parental separation, household public assistance, single-parent household and residential instability. Measures of LMM included long-term unemployment (LTU), long-term sickness absence (LTSA) and disability pension. Estimates of risk of each LMM measure, between 2010 and 2016 were calculated as hazard ratios (HRs) with 95% confidence intervals (CIs), using a Cox regression analysis.

**Results:**

Those exposed to CA had an elevated risk for all measures of LMM. Manual workers with a history of household public assistance had the highest risk estimates compared to non-manual workers with no CAs [adjusted HR spanning from 1.59 (LTSA) to 2.50 (LTU)]. Regardless of occupational class, the risk of LMM grew higher with increasing number of CAs (e.g. adjusted HR of LMM in manual workers with 3+ CAs: 1.87, 95% CI: 1.81–1.94). These patterns persisted after adjustments for a range of confounders, including psychiatric and somatic morbidity. Last, we found a small but significant mediating effect of occupational class in the association between CA and LMM.

**Conclusions:**

Information on CAs are important determinants of LMM in young adults, and especially in manual workers.

## Introduction

Experiences of stressful or traumatic childhood experiences, often referred to as childhood adversity (CA), can negatively affect childhood development and consequently health. Studies from many Western countries have consistently shown that young adults with a history of CA have a greater risk for a multitude of problems, compared with their peers without CA. Examples include mental and somatic morbidity,^1^^,^^2^ violent offending,^3^ substance abuse^1^^,^^2^ premature death^4^ and suicidal behavior.^2^^,^^5^ Specific CAs that have been linked to negative health and social outcomes include parental separation, single parenthood, parental criminality and parental psychiatric morbidity.^1^^,^^6^

Less attention has been paid to CAs as potential determinants of life opportunities, including later education^7^ and employment.^8^ Some studies, often based on self-reported retrospective data, have linked CAs to low educational attainment,^9–11^ as well as lower attachment to the labor market,^7^^,^^8^^,^^11–13^ and further shown that CA is associated with an elevated risk for labor market marginalization (LMM).^11–14^ LMM, can be defined as severe problems in obtaining and retaining a job, and has during the last decades been a growing and serious public health challenge in general and particularly in young people.^15^ In Sweden, during the last 10 years, the unemployment rate has been over 20% among young adults 16–24 years of age, which is higher than the average unemployment rate in the European Union.^16^ Moreover, the sickness absence (SA) rates in Sweden have historically been high, in 2015 around 4% of the Swedish population was estimated to be on SA at any given time, which was more than twice as many as in the other countries.^17^ There has also been an increase in the incidence of disability pension (DP) in young individuals over the last decades: in Sweden, 8100 individuals aged 19–29 years were granted temporary DP in 2016, which was more than twice as many in the same age group receiving DP during 2002.^18^ In order to have a comprehensive picture, LMM can be conceptualized from a social insurance perspective, including long-term unemployment (LTU), long-term SA (LTSA) and DP.

Although some studies have shown associations between CAs and subsequent LMM in the general populations,^11^^,^^13^ whether these associations can be extended to young adults, who in fact are gainfully employed at baseline, is less known. As studies have shown that those with a history of CAs who are in employment are doing better health-wise than those who are outside the labor market, young people with a history of CAs who have some employment experience may be more resilient to future LMM. However, to date, this has not been examined in a population-based setting.

Studies examining the role of socioeconomic status (SES), in terms of education, in the association between CA and LMM have shown that CA often leads to low educational attainment,^9^ that in turn is a risk factor for LMM.^19^^,^^20^ Moreover, some studies have demonstrated that education may play an important role in the relationship between CA and LMM.^11^^,^^21^ For example, a recent study from the UK examining the impact of CAs and education on employment in adulthood suggested that education may play a pivotal role in mitigating the effects of adversity on employment success.^11^ Other measures of SES include occupational class, often categorized as manual- and non-manual workers.^22^ CAs have been associated with occupational class, such that the prevalence of CAs is higher in manual workers.^23^ Moreover, epidemiological studies on socioeconomic inequalities in LMM have consistently shown that manual workers have an elevated risk for unemployment,^24^ SA^25^ and DP.^26^ Still, studies examining whether there are differences between manual and non-manual workers in the CA and LMM relationship in young employees are lacking to date. According to a recent Finnish study based on self-reported data, both CA and low SES increased the risk of work disability.^12^ However, this study did not distinguish between CA types. More scientific knowledge is needed on the potential mediating role of occupational class in these associations, as this could provide crucial knowledge on the mechanisms leading from CAs to LMM.

The current study investigated associations between specific and cumulative CAs and subsequent LMM, conceptualized as LTU, LTSA and DP in young employees. We further examined whether the association was mediated by occupational class.

## Methods

### Study population

The study population was identified from the Longitudinal Integration Database for Health Insurance and Labor Market Studies (LISA). Included were all individuals born in Sweden, aged 19–29 years, residing in Sweden on 31 December 2009 (*n* = 1 074 160). We only included those employed, and for which parental information was available (*n* = 669 179). Those with incomplete or missing information on occupational class (*n* = 77 683) were excluded, as were individuals who were either long-term unemployed, on LTSA or DP during the years 2006–2009 (*n* = 34 703). The final cohort comprised 556 793 individuals.

We used the unique Swedish personal identity number to link information from six registers. The National Patient Register (NPR) includes information on inpatient care since 1987 and for specialized outpatient care since 2001. Diagnoses in NPR are coded according to the International Classification of Diseases version 10 (ICD-10). The Cause of Death Register comprises information on all deaths of Swedish residents since 1952. The Swedish Social Insurance Agency database covers data on dates of SA and DP from 1994 and onwards. Families were linked together through the Multi-Generation Register, which contains all known relationships between children and parents (born 1932 or later) since 1961. The Total Population Register holds information on place of residence. Finally, the LISA register includes information on age, sex and other demographic characteristics.

### Exposure—childhood adversity

The CAs (see Supplementary table S1 for definitions) were selected based on prior research demonstrating them to have significant adverse health and social implications.^1^^,^^13^ In total, we included seven CAs: parental death, parental mental disorder, parental somatic disease, parental separation, household public assistance, single-parent household and residential instability. Based on data availability, the first three CAs were measured between birth and age 18 years, whereas the remaining adversities were captured from 1985 or 1990 and onwards. We assessed cumulative exposure to the studied CAs, by summarizing the total number of adversities and categorizing them into 0, 1, 2 and 3 or more CAs. Each CA was weighted equally in the analyses.

### Mediator—occupational class

We used the Swedish Standard Classification of Occupations (SSYK), obtained from LISA in 2009,^27^ to categorize manual and non-manual employees.^28^ The SSYK system has 10 categories which were dichotomized into manual and non-manual workers following a simplified version of the classification by Thell,^28^ which is based on what the work entails, education required and supervision responsibility.

### Outcome—LMM

LMM during the follow-up period was measured as LTU, LTSA and DP. LTU and LTSA were defined as having >180 and >90 registered days annually, respectively. In Sweden, all residents aged 16–65 years, with income from work, unemployment benefits or student benefits are covered by the national SA insurance regime and can be sickness absent with benefits if unable to work due to disease or injury. For those employed, the employer usually pays for the first 14 days of a SA spell. Thus, data on most of the short SA spells are not available. For individuals aged 30–64 years, DP can be granted to those who due to disease or injury have a permanently impaired work capacity.^18^ Individuals between 19 and 29 years can be granted time-restricted DP if work capacity is reduced, or if compulsory education is not completed before 19 years of age. A dichotomous variable for DP during follow-up was created.

### Confounders

Measured in 2009, we considered a range of potential confounders (Supplementary table S1 and table 1), with known associations to both CA and LMM. Adjustments were made for age, sex, educational level, family situation and type of residential area measured at baseline. Last, we adjusted for psychiatric and somatic morbidity, measured 2006–2009.

**Table 1:** Selected cohort characteristics for the study population, by exposure to childhood adversity (CA), in employed individuals aged 19–29 years old residing in Sweden in 2009

	All	No CAs	Parental death	Parental mental disorder	Parental somatic disease	Parental separation	Public assistance	Single-parent household	Residential instability	Total number of CAs		
										1	2	3+
All (*n*, row percent)	556 793 (100)	300 111 (54)	5714 (1)	42 209 (8)	68 216 (12)	135 772 (24)	18 620 (3)	198 061 (36)	20 978 (4)	97 361 (17)	103 688 (19)	55 633 (10)
Sociodemographic factors^a^												
* *Sex												
Women	275 520 (49)	146 193 (49)	2803 (49)	21 232 (50)	33 637 (49)	68 532 (50)	9663 (52)	100 492 (51)	11 140 (53)	48 577 (50)	52 475 (51)	28 275 (51)
Men	281 273 (51)	153 918 (51)	2911 (51)	20 977 (50)	34 579 (51)	67 240 (50)	8957 (48)	97 569 (49)	9838 (47)	48 784 (50)	51 213 (49)	27 358 (49)
Educational level												
Compulsory school (<9 years)	36 811 (7)	12 083 (4)	528 (4)	5116 (12)	5315 (8)	13 615 (10)	3639 (20)	21 057 (11)	3263 (16)	7394 (8)	9831 (9)	7503 (13)
High school (10–12 years)	342 598 (62)	176 858 (59)	3552 (62)	27 294 (65)	41 833 (61)	88 148 (65)	12 332 (66)	131 564 (66)	13 155 (63)	61 370 (63)	68 217 (66)	36 153 (65)
College or university (>12 years)	177 384 (32)	111 170 (37)	1634 (29)	9799 (23)	21 068 (31)	34 009 (25)	2649 (14)	45 440 (23)	4560 (22)	28 597 (29)	25 640 (25)	11 977 (22)
Occupational class												
Manual workers	402 212 (72)	204 783 (68)	4283 (75)	33 460 (79)	49 762 (73)	105 215 (77)	15 922 (86)	156 587 (79)	17 041 (81)	72 146 (74)	80 562 (78)	44 721 (80)
Non-manual workers	154 581 (28)	95 328 (32)	1431 (25)	8749 (21)	18 454 (27)	30 557 (23)	2698 (14)	41 474 (21)	3937 (19)	25 215 (26)	23 126 (22)	10 912 (20)
* *Family situation												
Married/living with partner without children^b^	15 327 (3)	9423 (3)	163 (3)	967 (2)	2032 (3)	2990 (2)	409 (2)	3862 (2)	574 (3)	2506 (3)	2125 (2)	1273 (2)
Married/living with partner with children^b^	80 155 (14)	42 969 (14)	891 (16)	6997 (17)	10 665 (16)	19 455 (14)	2827 (15)	27 764 (14)	3113 (15)	14 165 (15)	14 460 (14)	8561 (15)
Single/divorced/separated/widowed without children^b^	403 808 (73)	218 639 (73)	4241 (74)	29 995 (71)	49 205 (72)	98 874 (73)	12 926 (69)	143 075 (72)	15 016 (72)	69 653 (72)	75 599 (73)	39 917 (72)
Single/divorced/separated/widowed with children^b^	8409 (2)	3118 (1)	114 (2)	1169 (3)	1229 (2)	2847 (2)	628 (3)	4403 (2)	595 (3)	1706 (2)	2076 (2)	1509 (3)
Children (≤20 years old) living at home	49 094 (9)	25 962 (9)	305 (5)	081 (7)	5085 (7)	11 606 (9)	1830 (10)	18 957 (10)	1680 (8)	9331 (10)	9428 (9)	4373 (8)
* *Type of residential area												
Big city area	218 132 (39)	111 720 (37)	2339 (41)	16 910 (40)	27 222 (40)	58 258 (43)	9302 (50)	82 936 (42)	9068 (4)	38 682 (40)	43 497 (42)	24 233 (44)
Intermediate (>90 000 inhabitants)	200 040 (36)	110 262 (37)	1969 (34)	15 084 (36)	24 090 (35)	47 176 (35)	5993 (32)	68 804 (35)	7728 (37)	34 336 (35)	36 120 (35)	19 322 (35)
Small (rural municipalities)	138 621 (25)	78 129 (26)	1406 (25)	10 215 (24)	16 904 (25)	30 338 (22)	3325 (18)	46 321 (23)	4182 (20)	24 343 (25)	24 071 (23)	12 078 (22)
Health-related factors^c^												
Psychiatric morbidity	22 935 (4)	9444 (3)	284 (5)	3007 (7)	3142 (5)	7535 (6)	1364 (7)	11 055 (6)	1504 (7)	4331 (4)	5408 (5)	3752 (7)
Somatic morbidity	296 979 (53)	155 049 (52)	3027 (53)	24 184 (57)	37 278 (55)	75 452 (56)	10 985 (59)	110 500 (56)	12 090 (58)	52 743 (54)	57 308 (55)	31 879 (57)

Absolute numbers and column percent, with the exception of the first row).

a In 2009.

b Living at home.

c In 2006–2009.

### Statistical analysis

Statistical analyses were conducted using SAS, v.9.4. We were interested in the first occurrence of each outcome (LTU, LTSA, DP) and calculated rates of each outcome (i.e. number of cases for first time occurrence of an outcome divided by total person-years at risk), overall and by CAs and occupational class. Rates are presented in Supplementary table S2. We assessed person-years at risk as living in Sweden from 1 January 2010 to the date until either first outcome, death, emigration or end of follow-up (31 December 2016). For the outcomes LTU and LTSA, an additional censoring due to DP was considered. We next estimated a series of Cox regression models, first by comparing non-manual and manual workers (non-manual workers comprising the reference group). Second, we compared non-manual and manual workers among those with no CA exposures (the reference group constituted non-manual workers with no CA exposure). Third, for each CA, we combined exposures of CA and occupational class for each outcome. For each CA, the reference group comprised non-manual workers with no exposure to that particular CA. Individuals in the reference group for the specific CA could potentially have experienced other CAs. Last, we examined cumulative CA exposure (Supplementary figures S1–S3).

To test the potential mediation of the effect of CA on LMM by occupational class, a mediation analysis was conducted using a SAS macro for causal mediation analysis with survival data, provided by Valeri and VanderWeele.^29^ This is an extension of the Baron and Kenny method,^30^ in which a regression model examining the association between the proposed mediator (M) and the independent variable (IV) is compared with a regression model examining the association between the dependent variable (DV) and the IV together with the proposed M. A counterfactual framework, which allows for interactions between the IV and M, is then used to compare the two models to estimate the direct effect of the IV on DV and the indirect effect of the IV on DV via the proposed M. Comparison of the magnitude of the direct and indirect effect allows for the estimation of the proportion of total effect that is mediated. The results are presented as the direct effect of CA on the studied outcomes, the indirect effect of CA on outcomes mediated by occupational class, and the estimated total effect representing the combined natural direct and indirect effect. The causal effects are presented as hazard ratios (HRs) with 95% confidence intervals (CIs). For a more detailed description of the applied method, please see the tutorial by Lange *et al*.^31^

### Sensitivity analysis

In separate analyses, we compared characteristics for those excluded with the individuals included in the study.

## Results

Of the 556 793 young employees included in the study, nearly half (46%) had experienced at least one CA, and 10% had experienced three or more adversities (table 1). Single-parent household was the most prevalent CA (36%), followed by parental separation (24%). Individuals exposed to CAs were more likely to have a lower educational level and to work in manual occupations compared to those with no history of CA (table 1).

During the follow-up, 23 777 (4.3%) individuals experienced LTU, and 40 975 (7.4%) were on LTSA. The number of individuals who retrieved DP was relatively low, 1234 (0.2%).

We found an association between all CAs and LMM, such that, regardless of occupational class, individuals exposed to the studied CAs had an elevated risk for all measures of LMM compared with individuals with no CA (table 2). Corresponding rates for these associations are presented in Supplementary table S2. Manual workers had higher HRs of LMM compared with non-manual workers across all CAs. Household public assistance was most strongly associated with LTU [adjusted HR (aHR) for manual workers: 2.50; 95% CI 2.34–2.67]. The CA with the second highest HR for LTU for manual workers was residential instability followed by single-parent household and parental mental disorder (table 2).

**Table 2:** Associations between childhood adversity (CA), occupational class and labor market marginalization outcomes among the 556 793 employees, aged 19–29 years old residing in Sweden in 2009

Childhood adversity (CA)	Long-term unemployment (>180 days) (LTU)		Long-term sickness absence (>90 days) (LTSA)		Disability pension (DP)	
	Model I^a^	Model II^b^	Model I^a^	Model II^b^	Model I^a^	Model II^b^
All^c^						
Ref (non-manual)	1 (Ref)	1 (Ref)	1 (Ref)	1 (Ref)	1 (Ref)	1 (Ref)
Manual	1.75 (1.69–1.81)	1.47 (1.42–1.53)	1.33 (1.30–1.36)	1.21 (1.18–1.25)	2.30 (1.96–2.70)	1.23 (1.03–1.47)
No CA^d^						
Ref (non-manual)	1 (Ref)	1 (Ref)	1 (Ref)	1 (Ref)	1 (Ref)	1 (Ref)
Manual	1.74 (1.66–1.83)	1.56 (1.47–1.65)	1.25 (1.21–1.29)	1.20 (1.16–1.25)	2.11 (1.67–2.67)	1.31 (1.00–1.72)
Specific CAs						
Parental death						
Ref (non-manual, no parental death)	1 (Ref)	1 (Ref)	1 (Ref)	1 (Ref)	1 (Ref)	1 (Ref)
Non-manual, parental death	1.16 (0.87–1.55)	1.10 (0.82–1.47)	1.29 (1.07–1.56)	1.26 (1.04–1.52)	N/A	N/A
Manual, parental death	1.96 (1.72–2.24)	1.50 (1.32–1.72)	1.38 (1.24–1.54)	1.17 (1.05–1.30)	3.03 (1.79–5.14)	1.57 (0.92–2.68)
Parental mental disorder						
Ref (non-manual, no parental mental disorder)	1 (Ref)	1 (Ref)	1 (Ref)	1 (Ref)	1 (Ref)	1 (Ref)
Non-manual, parental mental disorder	1.49 (1.34–1.66)	1.35 (1.21–1.51)	1.44 (1.33–1.55)	1.30 (1.21–1.41)	2.14 (1.34–3.41)	1.67 (1.05–2.66)
Manual, parental mental disorder	2.50 (2.38–2.64)	1.85 (1.75–1.96)	1.95 (1.87–2.03)	1.53 (1.46–1.59)	4.46 (3.58–5.56)	1.80 (1.41–2.28)
Parental somatic disease						
Ref (non-manual, no parental somatic disease)	1 (Ref)	1 (Ref)	1 (Ref)	1 (Ref)	1 (Ref)	1 (Ref)
Non-manual, parental somatic disease	1.30 (1.19–1.41)	1.26 (1.16–1.37)	1.11 (1.04–1.18)	1.09 (1.02–1.16)	1.17 (0.76–1.79)	1.15 (0.75–1.77)
Manual, parental somatic disease	2.06 (1.96–2.16)	1.64 (1.55–1.73)	1.54 (1.48–1.59)	1.33 (1.28–1.38)	2.81 (2.25–3.52)	1.45 (1.15–1.85)
Parental separation						
Ref (non-manual, no parental separation)	1 (Ref)	1 (Ref)	1 (Ref)	1 (Ref)	1 (Ref)	1 (Ref)
Non-manual, parental separation	1.38 (1.29–1.48)	1.30 (1.21–1.39)	1.26 (1.21–1.33)	1.19 (1.13–1.24)	1.53 (1.10–2.13)	1.21 (0.87–1.68)
Manual, parental separation	2.25 (2.15–2.35)	1.80 (1.71–1.89)	1.66 (1.61–1.72)	1.40 (1.35–1.45)	3.59 (2.94–4.38)	1.56 (1.25–1.95)
Household public assistance						
Ref (non-manual, no household public assistance)	1 (Ref)	1 (Ref)	1 (Ref)	1 (Ref)	1 (Ref)	1 (Ref)
Non-manual, household public assistance	2.36 (2.02–2.75)	2.03 (1.74–2.37)	1.73 (1.53–1.95)	1.52 (1.34–1.71)	2.34 (1.10–4.99)	1.53 (0.72–3.27)
Manual, household public assistance	3.42 (3.22–3.63)	2.50 (2.34–2.67)	2.13 (2.03–2.24)	1.59 (1.51–1.68)	5.47 (4.26–7.02)	1.91 (1.46–2.50)
Single-parent household						
Ref (non-manual, no single-parent household)	1 (Ref)	1 (Ref)	1 (Ref)	1 (Ref)	1 (Ref)	1 (Ref)
Non-manual, single-parent household	1.60 (1.50–1.70)	1.48 (1.39–1.58)	1.34 (1.28–1.39)	1.23 (1.18–1.29)	1.81 (1.34–2.44)	1.36 (1.00–1.84)
Manual, single-parent household	2.50 (2.39–2.60)	2.02 (1.92–2.12)	1.73 (1.68–1.79)	1.46 (1.41–1.51)	3.91 (3.18–4.81)	1.65 (1.31–2.09)
Residential instability						
Ref (non-manual, no residential instability)	1 (Ref)	1 (Ref)	1 (Ref)	1 (Ref)	1 (Ref)	1 (Ref)
Non-manual, residential instability	1.39 (1.18–1.63)	1.30 (1.10–1.53)	1.50 (1.35–1.67)	1.37 (1.23–1.53)	1.60 (0.75–3.41)	1.06 (0.50–2.25)
Manual, residential instability	2.74 (2.58–2.92)	2.09 (1.95–2.24)	2.01 (1.91–2.11)	1.54 (1.46–1.62)	5.08 (3.96–6.52)	1.82 (1.39–2.39)

a Model 1: Crude.

b Model 2: Adjusted for age, sex, educational level, family situation, type of residential area and psychiatric and somatic morbidity.

c All individuals regardless of CA history, where reference group includes non-manual workers with no CA.

d Individuals with no CA history, where reference group comprises non-manual workers.

Individuals with a history of CAs also had an elevated risk of LTSA (table 2). Highest HRs were observed for public assistance and residential instability [aHRs spanning from 1.52 (non-manual workers with a history of residential instability) to 1.59 (manual workers with household public assistance)].

Highest risk for DP was observed in manual-workers with a history of public assistance (aHR: 1.91; 95% CI 1.46–2.50). Except for parental death, all CAs entailed elevated risk of DP in manual workers. Among those with parental death, few individuals who experienced parental death retrieved DP during the follow-up period (in total 15 individuals of which all were manual workers). HRs were also elevated in non-manual workers; however, most of these HRs were not statistically significant.

Investigation of cumulative CA and LMM (Supplementary figures S1–S3), we found evidence of an increasing risk of all outcomes with increasing number of CAs, for both occupational class groups. For LTU, compared to non-manual employees with no CAs, manual workers with 3+ CAs had the most elevated risk [aHR of 2.37 (95% CI 2.24–2.52)]. For LTSA, risk estimates were slightly lower overall, however, the dose–response pattern remained. For both LTU and LTSA, differences between manual and non-manual workers were statistically significant. The dose–response pattern was also observed for DP, but group differences were not as pronounced as for the other outcomes. Still, non-manual workers with 3+ CAs had over a 2-fold elevated risk compared to the reference group [aHR: 2.24 (95% CI 1.71–2.93)].

In the mediation analysis (table 3), we decomposed the total effect of each CA on the outcomes into a direct and indirect effect (through occupational class). Results revealed that the total effect of the studied adversities, as well as cumulative CA on all LMM measures was partially mediated by occupational class. This was true for all studied CAs except for parental death. For the association between household public assistance and LTU, the total aHR of 2.17 was decomposed into a direct HR of 2.04 (95% CI 1.94–2.15) and an indirect HR of 1.06 (95% CI 1.06–1.07). The proportion mediated through occupational class was about 8%. When omitting parental death, for the studied adversities, the proportion of CA on LTU mediated by occupational class spanned from 6.5% (residential instability) to 11.2% (parental mental disorder). For LTSA, similar proportions were observed. Last, for outcome DP, occupational class appeared to mediate the studied associations to a small but statistically significant effect (table 3, last section).

**Table 3:** Mediation analysis investigating the association of childhood adversity with subsequent labor market marginalization, mediated by occupational class, in employees aged 19–29 years old residing in Sweden in 2009

CA	Direct effect (HR,^a^ 95% CI)	Indirect effect (HR,^a^ 95% CI)	Total effect (HR,^a^ 95% CI)	Percentage of total effect mediated by occupational class
	**Long-term unemployment (>180 days) (LTU)**			
Parental death	1.08 (0.96–1.22)	1.02 (1.02–1.03)	1.11 (0.98–1.25)	24.2^b^
Parental mental disorder	1.38 (1.33–1.44)	1.04 (1.04–1.04)	1.44 (1.38–1.50)	11.2 (9.9–12.9)
Parental somatic disease	1.14 (1.10–1.18)	1.01 (1.01–1.01)	1.16 (1.12–1.20)	8.9 (6.5–12.5)
Parental separation	1.31 (1.27–1.35)	1.03 (1.03–1.03)	1.35 (1.31–1.39)	9.9 (8.8–11.3)
Household public assistance	2.04 (1.94–2.15)	1.06 (1.06–1.07)	2.17 (2.06–2.28)	8.0 (7.3–8.7)
Single-parent household	1.49 (1.45–1.53)	1.04 (1.04–1.05)	1.55 (1.51–1.59)	9.8 (9.0–10.7)
Residential instability	1.60 (1.52–1.69)	1.03 (1.03–1.04)	1.66 (1.57–1.75)	6.5 (5.6–7.5)
Cumulative CA (continuous)	1.19 (1.18–1.20)	1.02 (1.02–1.02)	1.21 (1.20–1.22)	10.3 (9.4–11.4)
	**Long-term sickness absence (>90 days) (LTSA)**			
Parental death	1.05 (0.96–1.15)	1.02 (1.01–1.02)	1.07 (0.97–1.17)	24.9^b^
Parental mental disorder	1.35 (1.31–1.40)	1.03 (1.03–1.03)	1.39 (1.35–1.43)	8.3 (7.3–9.5)
Parental somatic disease	1.11 (1.08–1.15)	1.01 (1.01–1.01)	1.12 (1.09–1.16)	7.5 (5.4–10.7)
Parental separation	1.22 (1.19–1.25)	1.02 (1.02–1.02)	1.24 (1.22–1.27)	9.0 (7.9–10.5)
Household public assistance	1.52 (1.46–1.59)	1.04 (1.04–1.05)	1.58 (1.52–1.65)	9.1 (7.9–10.2)
Single-parent household	1.30 (1.27–1.32)	1.03 (1.03–1.03)	1.34 (1.31–1.36)	9.9 (8.9–11.1)
Residential instability	1.40 (1.35–1.47)	1.02 (1.02–1.02)	1.44 (1.38–1.50)	6.1 (5.1–7.2)
Cumulative CA (continuous)	1.13 (1.12–1.14)	1.01 (1.01–1.01)	1.15 (1.14–1.16)	9.6 (8.5–10.7)
	**Disability pension (DP)**			
Parental death	1.18 (0.71–1.97)	1.02 (1.01–1.02)	1.20 (0.72–2.00)	9.1^b^
Parental mental disorder	1.64 (1.40–1.92)	1.03 (1.02–1.04)	1.68 (1.44–1.97)	5.4 (3.3–8.5)
Parental somatic disease	1.22 (1.04–1.42)	1.01 (1.01–1.01)	1.23 (1.05–1.44)	4.4 (2.0–17.7)
Parental separation	1.41 (1.26–1.59)	1.02 (1.01–1.03)	1.44 (1.28–1.62)	5.5 (3.3–8.9)
Household public assistance	1.92 (1.57–2.35)	1.04 (1.03–1.06)	2.00 (1.64–2.45)	6.1 (3.8–9.5)
Single-parent household	1.54 (1.37–1.72)	1.03 (1.02–1.04)	1.58 (1.41–1.77)	6.3 (3.8–9.5)
Residential instability	1.73 (1.42–2.12)	1.03 (1.01–1.03)	1.77 (1.45–2.17)	3.9 (2.4–6.6)
Cumulative CA (continuous)	1.23 (1.18–1.29)	1.01 (1.01–1.02)	1.25 (1.20–1.30277)	5.8 (3.5–8.7)

Hazard ratios (HRs) with 95% confidence intervals (CIs).

a Adjusted for age, sex, family situation, type of residential area and psychiatric and somatic morbidity.

b Due to few cases, confidence interval has not been estimated.

Direct effect: CA → LMM.

Indirect effect: CA → occupational class → LMM.

Total effect: combined direct and indirect effect.

Sensitivity analyses (Supplementary tables S3–S5) revealed that individuals excluded were more likely to possess less education, to be males (77 683 individuals excluded due to missing on SES), and to have a psychiatric and/or somatic morbidity compared to those included in the study. They had slightly lower rates of SA due to common mental disorders (CMDs) compared to the final study sample.

## Discussion

### Summary of findings

By using a large cohort of young employees in Sweden, the current study assessed associations between CA and LMM, and the potential mediating role of occupational class. We found that those exposed to CAs had a higher risk for all measures of LMM. Highest estimates for LMM were observed among manual workers with a history of household public assistance. Regardless of occupational class, the risk of LMM grew higher with increasing number of CAs. Associations remained after adjustments for a range of confounders. Last, our mediation analyses showed small but significant mediating effects of occupational class on the association between CA and LMM.

Our results are consistent with earlier studies, showing that individuals exposed to CA have an elevated risk of LMM in terms of unemployment,^11^^,^^14^ SA^11^^,^^14^ and DP.^12^^,^^13^ These studies did not, however, examine young employees specifically. Our findings suggest that young employees growing up in a household on public assistance have a significantly higher risk of all measures of LMM. This is in line with a recent Finnish study on municipal employees showing an association between economic difficulties in the childhood family and subsequent DP.^32^ Among other adversities included in our study, parental mental disorder stood out at especially linked with all measures of LMM. Earlier studies have linked history of parental mental disorder to DP in offspring.^13^^,^^32^

Risk of LMM grew higher with increasing number of CA in both non-manual and manual workers. A similar dose–response pattern, although not distinguishing between different groups of occupational class, has been shown in prior studies for unemployment,^7^^,^^8^^,^^11^^,^^21^ SA^7^^,^^11^ and DP^11^^,^^13^ respectively. Our study extends earlier work on socioeconomic gradients in CA and the studied LMM measures^11^^,^^12^^,^^21^ by considering the role of occupational class. Our findings suggest that the contribution of CAs on LMM may vary according to occupational class. Compared to non-manual workers, those employed in manual occupations had a markedly elevated risk for all measures of LMM. Among those exposed to CA, manual workers had a significantly higher risk of LMM compared to non-manual workers exposed to CA. Occupational class also acted as a mediator in the CA–LMM association. We are not aware of previous studies examining this in a nationwide setting. Somewhat in line with our findings, a recent Finnish study demonstrated an additive combined effect of CA and low adult SES on DP.^12^ That study did not, however, differentiate between types of CAs.

The pathways behind the association between CA and LMM have not been widely investigated. From a life-course perceptive, there may be different pathways through which stressful childhood events lead to morbidity and subsequently LMM in terms of SA and DP.^33^ For example, numerous studies have linked CA to psychiatric morbidity, that in turn is a strong risk factor for unemployment, SA and DP, especially among young individuals.^34^ A life-course approach further provides different conceptual models to explain associations between early life adversity and adult health.^33^ Our findings linking both CA and occupational class to LMM support the cumulative model, which assumes that both childhood and adulthood conditions are important to adult health and occupational achievements.^33^ In a dose–response manner, the risk of all measures of LMM increased with rising number of CAs, and accumulation of risk across the life span has been suggested as one etiologic pathway in a life course approach to persistent health problems.^33^ Our findings of a mediating effect of occupational class between CA and LMM is also in accordance with the pathway model in life-course epidemiology, which proposes an indirect effect of a third factor (i.e. occupational class) linking CA to LMM.^33^

Biological and psychological explanations to the association between CA and negative health outcomes have stated that exposure to CA is a major contributor to increased stress levels in childhood, with changes persisting across the life-span.^35^ This is turn can have effects on the individual’s developmental trajectory, with lifelong consequences for educational attainment, economic productivity, and labor market participation,^36^ and also within social and economic contexts. For example, studies have shown that wage inequities and precarious work are more common among individuals with a history of CA.^14^

Our study gives further support to the research underlining the importance of childhood environment for labor market attachment later in life, and our findings have important implications for prevention and intervention. The fact that CAs often co-occur has important implications for intervention, as prevention of single CAs among individuals exposed to several is unlikely to have effects. From a practice perspective, detecting CAs early is crucial in order to determine whether children exposed to stressful environments are on a trajectory that could lead to increased risk for later LMM. Besides increased recognition of CAs, extended support to children growing up in adversity should be part of any strategy aiming for reducing the effects they have on labor market outcomes. Last, our findings of occupational class acting as a mediator through which CA translates into risk for LMM call for further attention with regard to vocational intervention and prevention, as the impact of CA on LMM appears to be particularly detrimental in manual workers.

### Strengths and limitations

The strengths of this study include the population-based, longitudinal design and use of registers of high quality and completeness. The few existing previous studies on CA and LMM have often been retrospective and based on self-reported information, entailing risk for incorrect answers due to e.g. poor recall or blocking of certain memories.^37^ Furthermore, the large population size allowed for detailed stratified analyses, and ability to adjust for a range of important confounders. Nevertheless, there are several limitations that need to be discussed. The range of CAs is far from exhaustive and we do not assess the severity, duration or sequencing of any of these adversities. However, both the consistency of our results with other studies and the large cohort with high-quality data lends confidence to the validity of our findings. The lack of data on occupational class throughout the entire follow-up period might have led to over- or underestimation of the reported estimates. Moreover, there is a potential risk of unmeasured residual confounding as morbidity was only measured by specialized health care. An important aspect that we were not able to consider due to a lack of data, is ethnicity. Some studies have shown that patterns of CA may be differentially distributed across race,^38^ and future studies examining this with respect to LMM is warranted. In 2008, considerable changes in the social insurance regulations were implemented in Sweden, leading to stricter rules regarding granting of DP and SA.^39^ Our findings should be interpreted in the light of these considerations. Last, the findings should be interpreted in the light of the exclusion criteria and are generalizable the countries with comparable social insurance and healthcare systems.

## Conclusion

In conclusion, our study indicated that CA was associated with LTU, LTSA and DP in young employees in Sweden, and that occupational class plays an important role in this association, such that manual workers with a history of CA are especially vulnerable to LMM. Information on CAs may increase the understanding of determinants of LMM in young adults, and especially in manual workers.

## Supplementary Material

ckad019_Supplementary_Data

## Data Availability

The data in this study cannot be made available in the manuscript, the supplemental files, or a public repository. According to the Swedish Ethical Review Act, the Personal Data Act and the Administrative Procedure Act, data can only be made available after a legal review for researchers who meet the criteria for access to this type of sensitive and confidential data. Key points In studies on the long-term health and social consequences following exposure to childhood adversities (CAs), less attention has been paid to CAs as potential determinants of life opportunities, including later education and employment. A few studies have shown that CAs are associated with an elevated risk for labor market marginalization (LMM), defined as severe problems in obtaining and retaining a job. During the last decades, LMM has been a growing and serious public health challenge in general and particularly in young people. Our findings showed that those exposed to CAs had an elevated risk of LMM, in terms of long-term unemployment, long-term sickness absence and disability pension. The effects of CAs on LMM were partly mediated by occupational class. Information on CAs are important determinants of LMM in young adults, and especially in manual workers.
