# Supplementary material for: Childhood adversity and risk of later labor market marginalization in young employees in Sweden
Source: Eur J Public Health. 2023 Feb 24;33(2):264–71. doi: 10.1093/eurpub/ckad019 (PMC10066470; doi:10.1093/eurpub/ckad019)
Supplement: ckad019_Supplementary_Data [file ckad019_supplementary_data.zip › ckad019_Supplementary_Data/ejph-2020-07-om-0927-File005.docx]

**Supplementary material**

***Supplementary Figure 1.*** *Hazard ratios (HR) with 95% confidence intervals (CI) for long-term unemployment by cumulative childhood adversity (CA) and occupational class in employees aged 19-29 years old residing in Sweden in 2009. Model II was adjusted for age, sex, educational level, family situation, type of residential area and psychiatric and somatic morbidity at baseline. Non-manual workers with no CA provide the reference group.*

***Supplementary Figure 2.*** *Hazard ratios (HR) with 95% confidence intervals (CI) for long-term sickness absence by cumulative childhood adversity (CA) and occupational class in employees aged 19-29 years old residing in Sweden in 2009. Model II was adjusted for age, sex, educational level, family situation, type of residential area and psychiatric and somatic morbidity at baseline. Non-manual workers with no CA provide the reference group.*

***Supplementary Figure 3.*** *Hazard ratios (HR) with 95% confidence intervals (CI) for disability pension by cumulative childhood adversity (CA) and occupational class in employees aged 19-29 years old residing in Sweden in 2009. Model II was adjusted for age, sex, educational level, family situation, type of residential area and psychiatric and somatic morbidity at baseline. Non-manual workers with no CA provide the reference group.*

***Supplementary Table 1.*** *Definitions and classification of childhood adversity and psychiatric and somatic morbidity*

| **Childhood adversity** | **Definition** | **ICD Classification** | **Data Source (years)** |
| --- | --- | --- | --- |
| Parental death | Parental death | N/A | Causes of Death Register (1980-2008) |
| Parental mental disorder | Parental hospitalization for mental disorder | ICD-9: 290-319 | National Patient Register (1980-2008) |
| Parental somatic disease | Parental hospitalization for any of the conditions included in the Charlson Comorbidity Index ^40^ | See Brusselaers *et al*. ^40^ for complete code list | National Patient Register (1980-2008) |
| Parental separation | Having separated parents | N/A | LISA^1^ (1990-2008) |
| Household living on public assistance | Public assistance during at least one year, where more than 50 percent of the yearly income constituted public assistance | N/A | LISA^1^ (1990-2008) |
| Single-parent household | Single-parent household | N/A | LISA^1^ (1990-2008) |
| Residential instability | Three or more changes in place of residence | N/A | Total Population Register (1985-2008) |
|  |  |  |  |
| Psychiatric morbidity | Inpatient or specialized outpatient care with a psychiatric diagnosis | ICD-10^2^: F00-F99 | National Patient Register (2006-2009) |
| Somatic morbidity | Inpatient or specialized outpatient care with a diagnosis for somatic disorder | ICD-10^2^ chapter except for F00-F99, O80 and R00-R99 | National Patient Register (2006-2009) |

^1^ Longitudinal Integration Database for Health Insurance and Labor Market Studies

^2^ International Classification of Diseases

***Supplementary Table 2.*** *Rates between childhood adversity (CA), occupational class, and labor market marginalization outcomes among the 556,793 employees, aged 19-29 years old residing in Sweden in 2009^a^*

| **Childhood adversity (CA)** | **Long-term unemployment (>180 days) (LTU)^b^** | **Long-term sickness absence (>90 days) (LTSA)^b^** | **Disability Pension (DP)^b^** |
| --- | --- | --- | --- |
| All | 23,777 (63.1) | 40,975 (109.7) | 1,234 (3.2) |
| Non-manual workers | 4,300 (40.9) | 9,222 (88.5) | 176 (1.6) |
| Manual workers | 19,477 (71.7) | 31,753 (117.8) | 1,058 (3.8) |
|  |  |  |  |
| No CA | 10,392 (50.9) | 18,846 (93.0) | 478 (2.3) |
| Non-manual workers | 2,196 (33.7) | 5,132 (79.6) | 86 (1.3) |
| Manual workers | 8,196 (59.0) | 13,714 (99.3) | 392 (2.8) |
|  |  |  |  |
| Specific CAs |  |  |  |
| Parental death |  |  |  |
| All | 277 (71.8) | 459 (120.2) | 15 (3.8) |
| Non-manual workers | 46 (47.2) | 109 (113.8) | 0 (0.0) |
| Manual workers | 231 (80.2) | 350 (122.3) | 15 (5.0) |
|  |  |  |  |
| Parental mental disorder |  |  |  |
| All | 2,584 (91.6) | 4,438 (159.3) | 180 (6.2) |
| Non-manual workers | 351 (59.4) | 722 (124.0) | 20 (3.3) |
| Manual workers | 2,233 (100.1) | 3,716 (168.6) | 160 (6.9) |
|  |  |  |  |
| Parental somatic disease |  |  |  |
| All | 3,368 (73.2) | 5,655 (124.1) | 181 (3.8) |
| Non-manual workers | 640 (51.2) | 1,202 (97.0) | 24 (1.9) |
| Manual workers | 2,728 (81.5) | 4,453 (134.3) | 157 (4.5) |
|  |  |  |  |
| Parental separation |  |  |  |
| All | 7,140 (78.3) | 11,966 (132.5) | 438 (4.7) |
| Non-manual workers | 1,084 (52.5) | 2,171 (106.4) | 48 (2.3) |
| Manual workers | 6,056 (85.9) | 9,795 (140.2) | 390 (5.4) |
|  |  |  |  |
| Household public assistance |  |  |  |
| All | 1,612 (131.3) | 2,213 (181.4) | 104 (8.1) |
| Non-manual workers | 170 (94.8) | 268 (151.2) | 7 (3.8) |
| Manual workers | 1,442 (137.6) | 1,945 (186.5) | 97 (8.8) |
|  |  |  |  |
| Single-parent household |  |  |  |
| All | 10,849 (81.6) | 17,680 (134.2) | 645 (4.7) |
| Non-manual workers | 1,580 (56.3) | 3,008 (108.5) | 70 (2.4) |
| Manual workers | 9,269 (88.3) | 14,672 (141.0) | 575 (5.3) |
|  |  |  |  |
| Residential instability |  |  |  |
| All | 1,411 (101.2) | 2,301 (167.0) | 104 (7.2) |
| Non-manual workers | 149 (56.3) | 341 (131.1) | 7 (2.6) |
| Manual workers | 1,262 (111.8) | 1,960 (175.4) | 97 (8.2) |

*^a^ Rates per 10,000 person-years, number of cases divided by the total person-years at risk, related to each of the three outcomes.*

*^b^ Absolute numbers and rates within parenthesis. E.g. 23,777 of the 556,793 individuals experienced LTU during the follow-up period with a corresponding rate of 63.1 per 10,000 individuals.*

***Supplementary Table 3.*** *Cohort characteristics comparing the 556,793 individuals included with the 77,783 individuals excluded due to missing on occupational class and the 34,703 individuals excluded due to LMM in 2006-2009. Numbers presented as row percentages.*

| **Cohort characteristics^1^** | **Included** | **Excluded due to missing on occupational class** | **Excluded due to LMM in 2006-2009** |
| --- | --- | --- | --- |
| All, (n, row percent) | 556,793 (100%) | 77,683 (100%) | 34,703 (100%) |
| ***Sociodemographic factors*** |  |  |  |
| Sex |  |  |  |
| Women | 49% | 41% | 52% |
| Men | 51% | 60% | 52% |
|  |  |  |  |
| Education (years) |  |  |  |
| Compulsory school (<9) | 7% | 11% | 18% |
| High school (10–12) | 62% | 73% | 68% |
| College or university (>12) | 32% | 16% | 18% |
|  |  |  |  |
| Family situation |  |  |  |
| Married/living with partner without children^2^ | 3% | 1% | 3% |
| Married/living with partner with children^2^ | 14% | 4% | 22% |
| Single/divorced/separated/widowed without children^2^ | 73% | 55% | 73% |
| Single/divorced/separated/widowed with children^2^ | 2% | 1% | 4% |
| Children (≤20 years old)^2^ | 9% | 39% | 1% |
|  |  |  |  |
| Type of residential area |  |  |  |
| Big city area | 39% | 37% | 34% |
| Intermediate (>90,000 inhabitants) | 36% | 35% | 40% |
| Small (rural municipalities) | 25% | 28% | 29% |
| Health-related factors^3^ |  |  |  |
| Psychiatric morbidity | 4% | 6% | 19% |
| Somatic morbidity | 53% | 54% | 72% |

^1^ In 2009 ^2^ Living at home ^3^ In 2006-2009

***Supplementary Table 4.*** *Associations between childhood adversity (CA), and labor market marginalization among employees aged 19-29 years residing in Sweden in 2009. Multi-adjusted Hazard ratios^a^ (aHRs) with 95% confidence intervals (CIs)*

|  | **Excluded due to missing on occupational class (n=77,683)** | **Excluded due to LMM in 2006-2009 (n=34,703)** |
| --- | --- | --- |
|  |  |  |
| **Childhood adversity (CA)** | **Long-term unemployment (>180 days) (LTU)** | |
| No CA | 1 (REF) | 1 (REF) |
| Parental death | 1.02 (0.77-1.34) | 1.11 (0.90-1.37) |
| Parental mental disorder | 1.30 (1.19-1.43) | 1.12 (1.04-1.21) |
| Parental somatic disease | 1.08 (0.99-1.18) | 1.05 (0.97-1.12) |
| Parental separation | 1.28 (1.20-1.37) | 1.13 (1.07-1.20) |
| Household public assistance | 1.95 (1.76-2.15) | 1.29 (1.18-1.42) |
| Single-parent household | 1.51 (1.42-1.60) | 1.20 (1.14-1.26) |
| Residential instability | 1.38 (1.23-1.54) | 1.24 (1.12-1.37) |
|  |  |  |
|  | **Long-term sickness absence (>90 days) (LTSA)** | |
| No CA | 1 (REF) | 1 (REF) |
| Parental death | 0.68 (0.50-0.92) | 1.09 (0.91-1.30) |
| Parental mental disorder | 1.30 (1.20-1.42) | 1.09 (1.02-1.16) |
| Parental somatic disease | 1.13 (1.05-1.23) | 1.11 (1.05-1.17) |
| Parental separation | 1.16 (1.10-1.23) | 1.09 (1.04-1.14) |
| Household public assistance | 1.31 (1.18-1.46) | 1.13 (1.04-1.23) |
| Single-parent household | 1.26 (1.19-1.34) | 1.02 (0.98-1.07) |
| Residential instability | 1.19 (1.07-1.32) | 1.04 (0.96-1.14) |
|  |  |  |
|  | **Disability pension (DP)** | |
| No CA | 1 (REF) | 1 (REF) |
| Parental death | 1.13 (0.64-2.00) | 0.85 (0.55-1.30) |
| Parental mental disorder | 0.91 (0.74-1.13) | 0.95 (0.83-1.09) |
| Parental somatic disease | 1.13 (0.93-1.37) | 1.03 (0.91-1.17) |
| Parental separation | 0.95 (0.82-1.10) | 0.92 (0.83-1.02) |
| Household public assistance | 1.02 (0.80-1.32) | 0.78 (0.64-0.97) |
| Single-parent household | 1.05 (0.91-1.22) | 0.78 (0.71-0.86) |
| Residential instability | 1.16 (0.91-1.47) | 0.80 (0.65-0.98) |

^a^ Adjusted for age, sex, educational level, family situation, type of residential area and psychiatric and somatic morbidity

***Supplementary Table 5.*** *Associations between cumulative childhood adversity (CA), and labor market marginalization among employees aged 19-29 years residing in Sweden in 2009. Multi-adjusted Hazard ratios^a^ (aHRs) with 95% confidence intervals (CIs)*

|  | **Excluded due to missing on occupational class (n=77,683)** | **Excluded due to LMM in 2006-2009 (n=34,703)** |
| --- | --- | --- |
|  |  |  |
| **Childhood adversity (CA)** | **Long-term unemployment (>180 days) (LTU)** | |
| No CA | 1 (REF) | 1 (REF) |
| 1 CA | 1.35 (1.24-1.47) | 1.11 (1.03-1.19) |
| 2 CAs | 1.43 (1.32-1.55) | 1.19 (1.11-1.27) |
| 3+ CAs | 1.79 (1.65-1.96) | 1.31 (1.22-1.42) |
|  |  |  |
|  | **Long-term sickness absence (>90 days) (LTSA)** | |
| No CA | 1 (REF) | 1 (REF) |
| 1 CA | 1.24 (1.15-1.34) | 1.05 (0.99-1.12) |
| 2 CAs | 1.29 (1.20-1.39) | 1.07 (1.01-1.13) |
| 3+ CAs | 1.44 (1.32-1.56) | 1.15 (1.08-1.22) |
|  |  |  |
|  | **Disability pension (DP)** | |
| No CA | 1 (REF) | 1 (REF) |
| 1 CA | 1.06 (0.87-1.29) | 0.82 (0.72-0.93) |
| 2 CAs | 0.97 (0.81-1.17) | 0.85 (0.75-0.95) |
| 3+ CAs | 1.12 (0.91-1.37) | 0.75 (0.65-0.86) |

^a^ Adjusted for age, sex, educational level, family situation, type of residential area and psychiatric and somatic morbidity
