# Supplementary figures and images for: Childhood adversity and risk of later labor market marginalization in young employees in Sweden
Source: Eur J Public Health. 2023 Feb 24;33(2):264–71. doi: 10.1093/eurpub/ckad019 (PMC10066470; doi:10.1093/eurpub/ckad019)

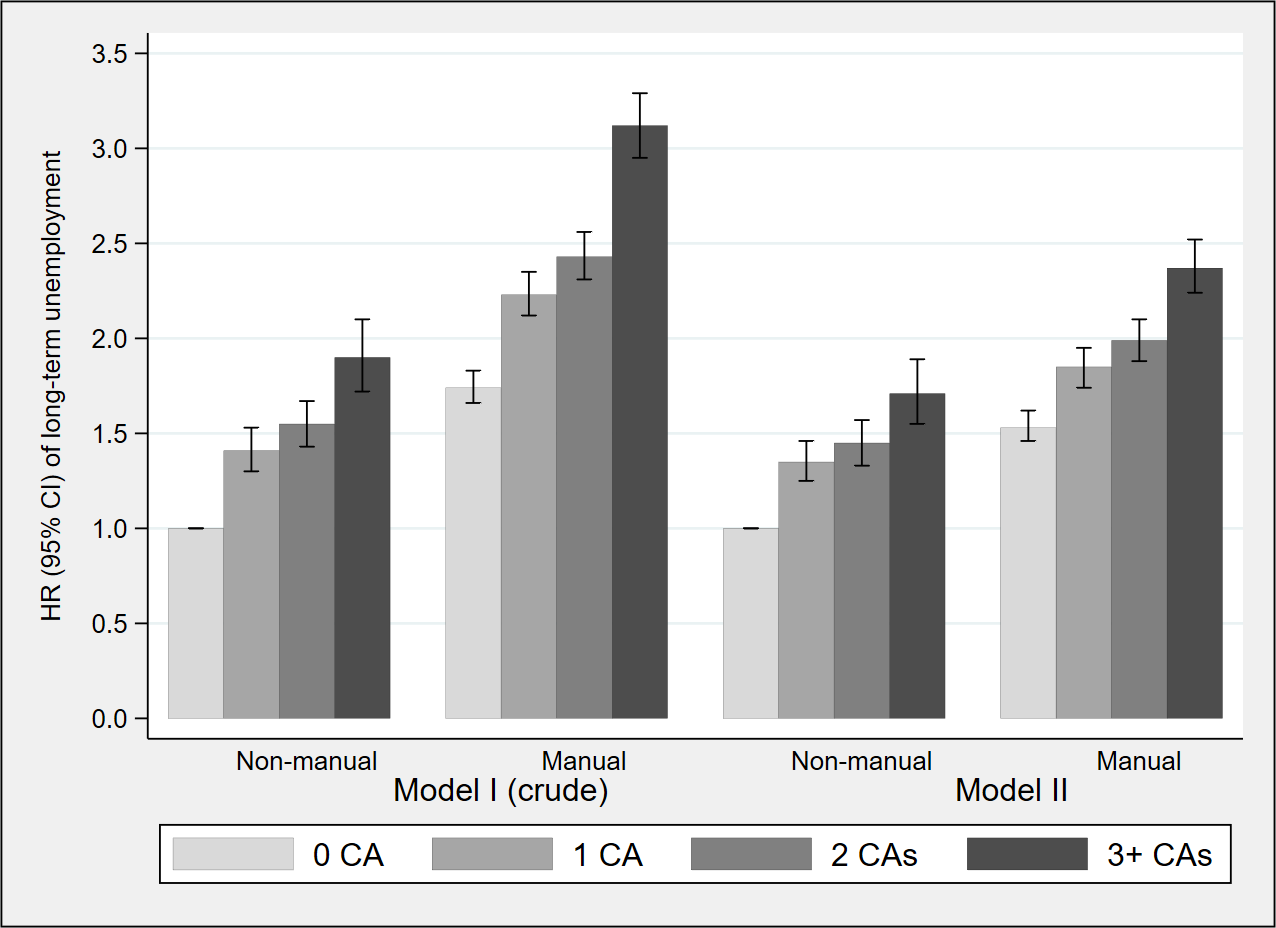

Supplement: ckad019_Supplementary_Data [file ckad019_supplementary_data.zip › ckad019_Supplementary_Data/ejph-2020-07-om-0927-File002.tiff]

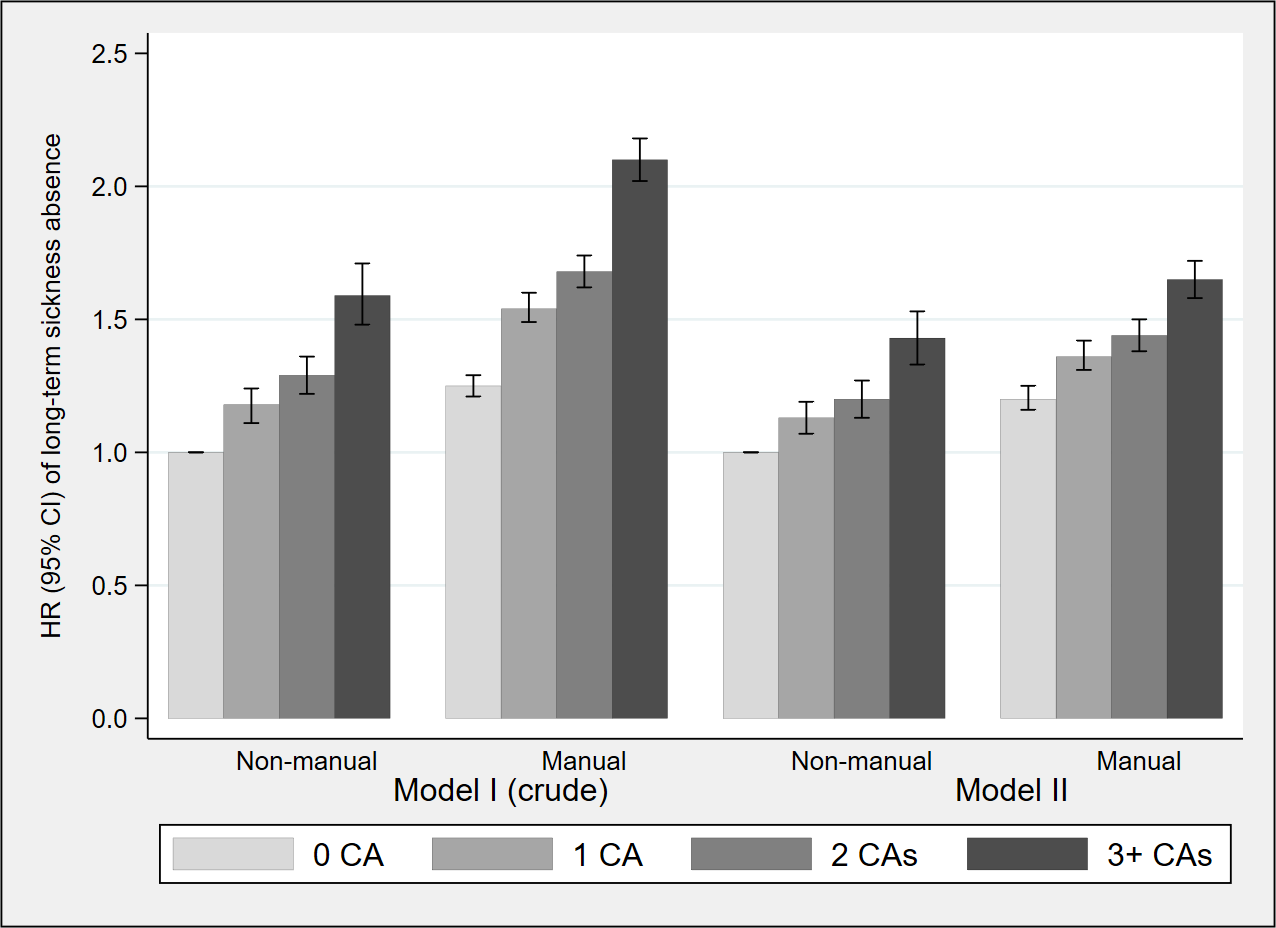

Supplement: ckad019_Supplementary_Data [file ckad019_supplementary_data.zip › ckad019_Supplementary_Data/ejph-2020-07-om-0927-File003.tiff]

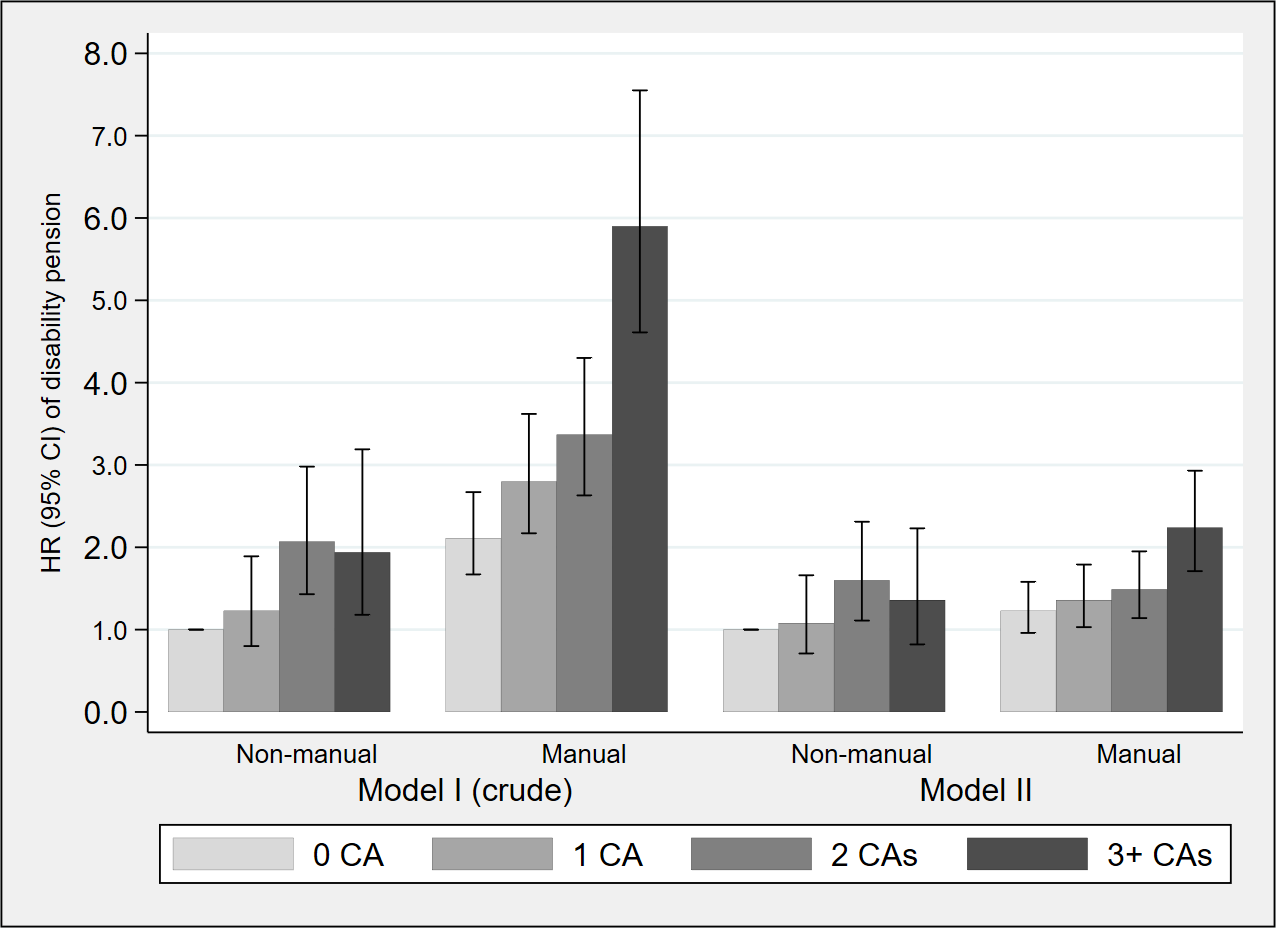

Supplement: ckad019_Supplementary_Data [file ckad019_supplementary_data.zip › ckad019_Supplementary_Data/ejph-2020-07-om-0927-File004.tiff]
